# Supplementary material for: Association between oral microbiome and seven types of cancers in East Asian population: a two-sample Mendelian randomization analysis
Source: Front Mol Biosci. 2023 Nov 21;10:1327893. doi: 10.3389/fmolb.2023.1327893 (PMC10702768; doi:10.3389/fmolb.2023.1327893)
Supplement: Supplementary file 1 [file DataSheet1.ZIP › Supplementary Materials/MR plots for tongue/tongue═╝/Ovarian cancer/pheno.1312_to_ovarian cancer_leave_one_out.pdf]

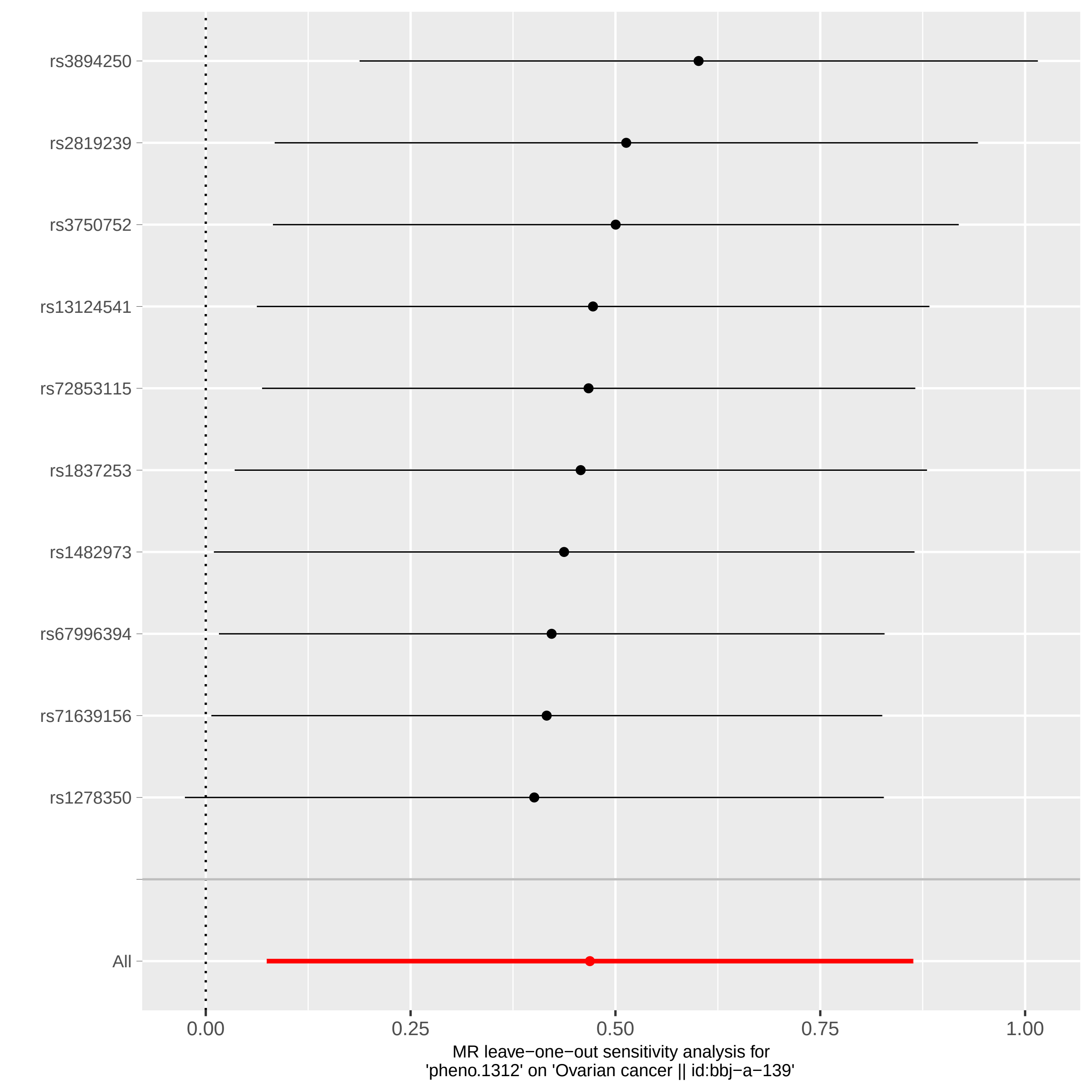
rs3894250
rs2819239
rs3750752
rs13124541
rs72853115
rs1837253
rs1482973
rs67996394
rs71639156
rs1278350
All
0.00
0.25
0.50
0.75
1.00
MR leave−one−out sensitivity analysis for
'pheno.1312' on 'Ovarian cancer || id:bbj−a−139'
